# Theanine, a tea-plant-specific non-proteinogenic amino acid, is involved in the regulation of lateral root development in response to nitrogen status

**DOI:** 10.1093/hr/uhac267

**Published:** 2022-12-02

**Authors:** Tingting Chen, Shijia Lin, Ziping Chen, Tianyuan Yang, Shupei Zhang, Jinsong Zhang, Guohua Xu, Xiaochun Wan, Zhaoliang Zhang

**Affiliations:** State Key Laboratory of Tea Plant Biology and Utilization, Anhui Agricultural University, Hefei 230036, China; State Key Laboratory of Tea Plant Biology and Utilization, Anhui Agricultural University, Hefei 230036, China; State Key Laboratory of Tea Plant Biology and Utilization, Anhui Agricultural University, Hefei 230036, China; State Key Laboratory of Tea Plant Biology and Utilization, Anhui Agricultural University, Hefei 230036, China; State Key Laboratory of Tea Plant Biology and Utilization, Anhui Agricultural University, Hefei 230036, China; State Key Laboratory of Tea Plant Biology and Utilization, Anhui Agricultural University, Hefei 230036, China; State Key Laboratory of Crop Genetics and Germplasm Enhancement, Nanjing Agricultural University, Nanjing 210095, China; State Key Laboratory of Tea Plant Biology and Utilization, Anhui Agricultural University, Hefei 230036, China; State Key Laboratory of Tea Plant Biology and Utilization, Anhui Agricultural University, Hefei 230036, China

## Abstract

Glutamine synthetase type I (GSI)-like proteins are proposed to mediate nitrogen signaling and developmental fate by synthesizing yet unidentified metabolites. Theanine, the most abundant non-proteinogenic amino acid in tea plants, is the first identified metabolite synthesized by a GSI-like protein (CsTSI) in a living system. However, the roles of theanine in nitrogen signaling and development are little understood. In this study we found that nitrogen deficiency significantly reduced theanine accumulation and increased lateral root development in tea plant seedlings. Exogenous theanine feeding significantly repressed lateral root development of seedlings of tea plants and the model plant *Arabidopsis*. The transcriptomic analysis revealed that the differentially expressed genes in the roots under theanine feeding were enriched in the apoplastic pathway and H_2_O_2_ metabolism. Consistently, theanine feeding reduced H_2_O_2_ levels in the roots. Importantly, when co-treated with H_2_O_2_, theanine abolished the promoting effect of H_2_O_2_ on lateral root development in both tea plant and *Arabidopsis* seedlings. The results of histochemical assays confirmed that theanine inhibited reactive oxygen species accumulation in the roots. Further transcriptomic analyses suggested the expression of genes encoding enzymes involved in H_2_O_2_ generation and scavenging was down- and upregulated by theanine, respectively. Moreover, the expression of genes involved in auxin metabolism and signaling, cell division, and cell expansion was also regulated by theanine. Collectively, these results suggested that CsTSI-synthesized theanine is likely involved in the regulation of lateral root development, via modulating H_2_O_2_ accumulation, in response to nitrogen levels in tea plants. This study also implied that the module consisting of GSI-like protein and theanine-like metabolite is probably conserved in regulating development in response to nitrogen status in plant species.

## Introduction

The tea plant (*Camellia sinensis*) is a vital economic crop around the world. It synthesizes abundant secondary metabolites that determine the sensory quality and health benefits of tea infusion. Among these metabolites, theanine (γ-glutamylethylamide) is the most characteristic one. It is a tea-plant-specific non-proteinogenic amino acid, accounting for ~50% of the free amino acids and 1–6% of dry weight of tender shoots [1,2]. Since theanine greatly enhances the distinctive umami taste and anti-stress effects of tea, its content highly correlates with the quality of green teas [3]. Theanine is mainly synthesized and stored in the roots of tea plants in winter, and moves the long distance from roots to new shoots in spring [4,5]. However, the physiological and developmental roles of theanine in tea plants are largely unknown.

Theanine synthetase (CsTSI) synthesizes theanine from glutamate and ethylamine [6–8]. Phylogenetic analysis showed that CsTSI belongs to the glutamine synthetase type I (GSI)-like proteins [7]. GSI-like proteins are fusion proteins with a C terminal type I glutamine synthetase domain linked with a nodulin-like domain in the N terminal [9,10]. The first described GSI-like gene, *fluG*, was characterized in the fungus *Aspergillus nidulans* as an early regulator of asexual sporulation under nitrogen (N) starvation [11]. The homologs of FluG are named as GSI-like and are widespread in higher plants [9]. NodGS, the GSI-like protein in *Arabidopsis*, was shown to regulate root development [12]. The GSI-like genes in *Medicago truncatula* are highly expressed in the lateral root (LR) primordium and nodule primordium, and were shown to be induced by externally applied amino acids, suggesting a role for these GSI-like genes in LR development and nodulation in response to N [13]. Although these GSI-like proteins are important for sporulation and root development, they do not have glutamine synthetase activity and are not responsible for glutamine synthesis [11–13]. Instead, they are proposed to synthesize some yet unidentified metabolite(s) linked to N signaling and developmental fate [12–14].

To our knowledge, theanine is the first identified metabolite synthesized by GSI-like in a living system. However, the role of theanine in N signaling and development in tea plants is unknown [5]. In animals, theanine alleviates bacterial-induced oxidative damage in the liver by upregulating the expression of genes encoding catalase (CAT), superoxide dismutase (SOD), and glutathione peroxidase (GPx), and it also prevents alcoholic liver injury by promoting the antioxidant ability of liver cells [15,16]. In tea plants, theanine accumulation and N level showed trends opposite to root development, while reactive oxygen species (ROS) accumulation was consistent with LR development [15–18]. The positive roles of ROS in root development have been extensively studied in plants [19]. However, whether theanine regulates root development has not been investigated.

The root system of tea plants undergoes a series of morphological changes during the growth period. It gradually develops from a primary root (PR) system to a fibrous root system. The morphological structure and the growth of roots directly affect water availability and mineral nutrient uptake of tea plants, and are thereby regulated by nutrient levels [16]. As a major part of the root system, LRs facilitate resource capture by tea plants. LRs originate from pericycle founder cells that subsequently differentiate into LR primordium, leading to LR meristem activation and the division of newly generated cells [20].

The formation and outgrowth of LRs rely on the intricate regulation of cell cycle and hormone signaling [21]. Auxin dominates throughout LR development. Exogenous auxin increases LR number and promotes the anticlinal division of pericycle cells [22]. In *Arabidopsis*, auxin transporter-like protein 3 (LAX3) induces the expression of cell-wall-remodeling genes, resulting in cell wall breakdown and therefore boosting LR emergence [17,23]. Auxin signaling mediated by Auxin/Indole-3-Acetic Acid (Aux/IAA) and auxin response factors (ARFs) regulates LR development by inducing the expression of *LATARAL ROOT PRIMORDIUM* (*LRP1*) in LR meristem [24]. The N supply regulates LR development. Excess N supply inhibits LR development [15]. When plants suffer from mild N deficiency, LR length is significantly increased via the cooperative regulation of N and auxin signaling [15]. Under severe N deficiency, LR length is reduced and LR initiation is impaired [25].

H_2_O_2_ also regulates LR development. Previous studies found that H_2_O_2_ treatment significantly increased LR density by activating LR pre-branch sites and LR primordia [26,27]. Conversely, treatment with an H_2_O_2_ production inhibitor, diphenylene iodonium (DPI), decreased LR density in *Arabidopsis* [26]. The RESPIRATORY BURST OXIDASE HOMOLOGS (RBOHs) and class III peroxidases mediate ROS production in extracellular spaces, and thus facilitate LR development by promoting cell wall softening [27,28].

In this study we explored the role of theanine in LR development and the underlying mechanism by pharmacological and molecular approaches, as well as transcriptomics analyses. We found that theanine impeded endogenous H_2_O_2_ accumulation and thereby inhibited LR development. Our results provide insights into the role of GSI-like synthesized theanine in the LR development of tea plants in response to N levels.

## Results

### Nitrogen deficiency decreased theanine accumulation and promoted lateral root development

**Figure 1:**
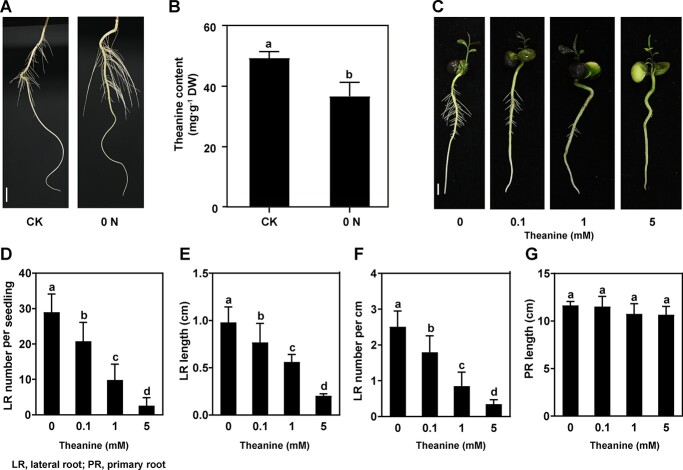
Theanine inhibited LR development of tea plant seedlings. (A) LR phenotypes of tea plant seedlings treated with normal N (CK) and N deficiency (0 N). Scale bar = 1 cm. (B) Theanine contents in the roots of tea plant seedlings under CK and 0 N. (C) LR phenotypes of tea plant seedlings grown in hydroponic solution containing 0, 0.1, 1, or 5 mM theanine for 2 weeks. Scale bar = 1 cm. (D–G) Number of emerged LRs (>1 mm) per seedling (D), LR length (E), density of emerged LRs (F), and PR length (G). Mean and standard error values were calculated from three independent experiments. Fifteen seedlings for each treatment were used for the statistical analyses shown in D–G. LR density (LR number per centimeter) was calculated using the formula LR number/length of PR. Different lowercase letters above the error bars indicate significant differences by one-way ANOVA and Duncan’s multiple range test (*P* < .05).

To study the role of theanine in the N-regulated LR development, we first verified LR development phenotype under normal (CK) and N deficiency (0 N) conditions. The results showed that 0 N promoted the growth of LRs (Fig. 1A). At the same time, theanine level in the roots was significantly decreased by 0 N (Fig. 1B). These results were consistent with previous observations [16–18]. In these previous studies, stem-cutting clone plants were used. Here we used seedlings from seeds. This means that these two types of tea plants respond similarly to N deficiency in terms of LR development and theanine accumulation. Therefore, we used these seedlings from seeds to study the role of theanine in the following experiments.

### Theanine inhibited lateral root development in tea plants and *Arabidopsis*

To explore the role of theanine in LR development of tea plants, we analyzed the root phenotypes of hydroponic tea plant seedlings treated with different concentrations of theanine (0, 0.1, 1, and 5 mM) for 14 days. We observed that, as the theanine concentration increased, LR growth was gradually repressed (Fig. 1C–F). LR number decreased by 40% when tea seedlings were exposed to 0.1 mM theanine and 66% in response to 1 mM theanine (*P* < .05) (Fig. 1D). Likewise, LR length was reduced by 22% and 43% when tea seedlings were treated with 0.1 and 1 mM theanine, respectively (Fig. 1E). Moreover, LR density also decreased significantly (Fig. 1F). The 5 mM theanine treatment almost completely impeded LR development (Fig. 1C–F). However, the growth of PRs was not obviously affected by theanine treatments (Fig. 1G). The above results suggested that theanine inhibits LR development in tea plants.

To validate the role of theanine in LR development, we further examined the effects of theanine in LR in the model plant *Arabidopsis*. Five-day-old seedlings were treated with 0, 0.01, 0.1, 1, and 5 mM theanine for 2 days. Results showed that LR development was also inhibited in a concentration-dependent manner (Supplementary Data Fig. S2). LR number and length declined by 44% and 32%, respectively, when the seedlings were exposed to 1 mM theanine (Supplementary Data Fig. S2B and C); meanwhile, LR density was significantly lower in seedlings treated with 1 mM theanine compared with the control (Supplementary Data Fig. S2D). There was no significant difference in PR length when the seedlings were treated with different concentrations of theanine (Supplementary Data Fig. S2E). These results suggested that theanine also inhibited LR development in the model plant *Arabidopsis*, and further supported the role of theanine in inhibiting LR development in tea plants.

### Differentially expressed genes in response to theanine treatment were enriched in extracellular region and H_2_O_2_ metabolic processes

**Figure 2:**
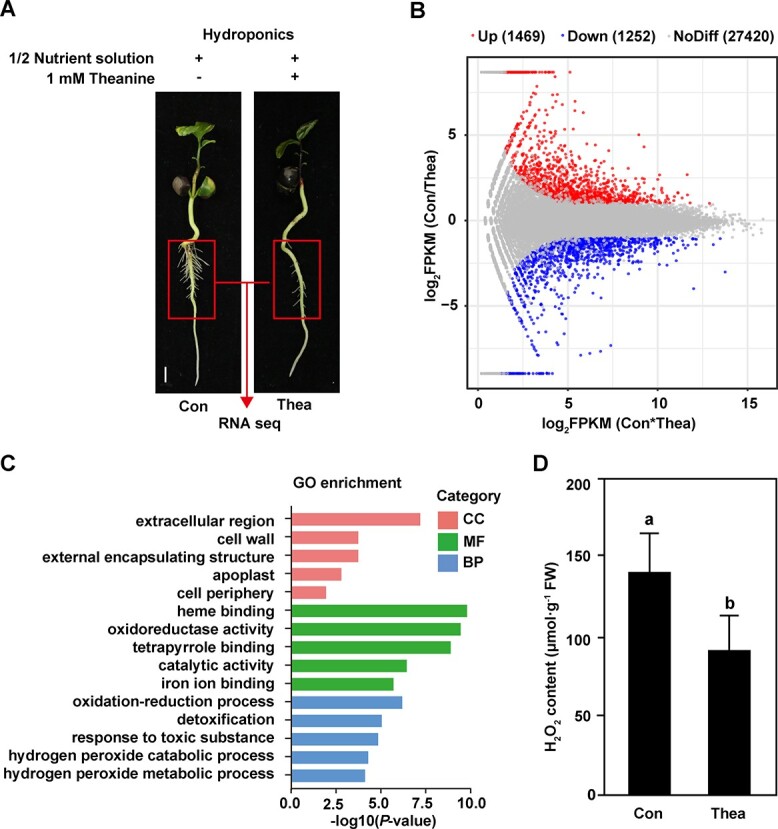
DEGs induced by theanine treatment and functional enrichment analysis. (A) Transcriptome sequencing samples. Scale bar = 1 cm. (B) MA plot of DEGs. Red and blue dots represent up- and downregulated genes, respectively. Gray dots represent non-significantly changed genes. (C) GO enrichment analysis of DEGs in response to theanine treatment. (D) H_2_O_2_ content in roots of tea plant seedlings under control (CK) and theanine treatment.

To elucidate the mechanism underlying the inhibiting role of theanine in LR development, we performed transcriptomic analyses of the roots of tea plant seedlings grown under control condition (CK) and 1 mM theanine (theanine) treatment (Fig. 2A). The DEGs, including 1469 upregulated and 1252 downregulated genes, in response to theanine were identified (Fig. 2B). Interestingly, GO enrichment analysis of these DEGs were significantly enriched in extracellular region, cell wall and apoplast for cellular component (CC), oxidoreductase activity and catabolic activity for molecular function (MF), and oxidation–reduction process, H_2_O_2_ catabolic process and H_2_O_2_ metabolic process for biological process (BP) (Fig. 2C). Furthermore, H_2_O_2_ content was reduced by 27% under theanine treatment compared with the control (Fig. 2D). It was reported that H_2_O_2_ deposited in the extracellular region/apoplast acts on cell wall remodeling to regulate LR emergence [26]. Therefore, these results implied that theanine modulates H_2_O_2_ accumulated to inhibit LR development.

### Theanine abolished the promoting effect of H_2_O_2_ on lateral root development

**Figure 3:**
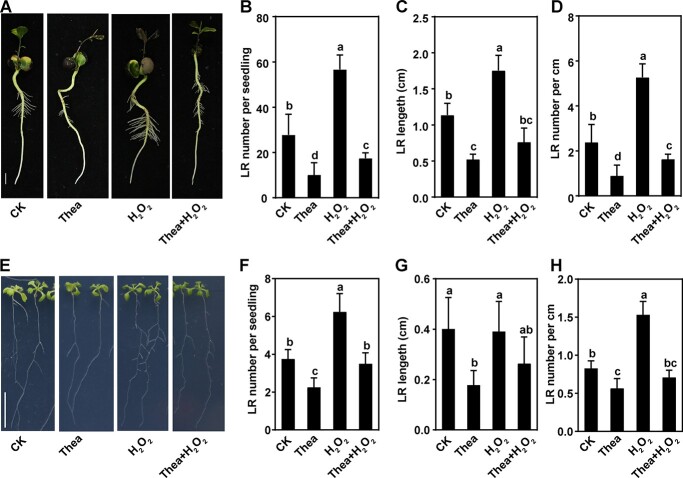
Theanine abolished the promoting effect of H_2_O_2_ on LR development in tea plants. Tea plant and *Arabidopsis* seedlings grew under control (CK), 1 mM theanine (Thea), 200 μM H_2_O_2_ (H_2_O_2_), or combination (Thea + H_2_O_2_) treatment. (A and E) Phenotypes of tea plant and *Arabidopsis* seedlings. Scale bar = 1 cm. (B and F) Number of emerged LRs per seedling. (C and G) LR length. (D and H) Emerged LR density. Mean and standard error values were from the results of three independent experiments. Fifteen seedlings for each treatment were used for the statistical analyses in B–D and F–H. Different lowercase letters above the error bars indicate significant differences by one-way ANOVA and Duncan’s multiple range test (*P* < .05).

To test our hypothesis that theanine inhibits LR development by modulating H_2_O_2_ accumulation, we analyzed the root phenotypes of hydroponic tea plant seedlings treated with or without 1 mM theanine, 200 μM H_2_O_2_, or the combination of 1 mM theanine and 200 μM H_2_O_2_ (Fig. 3A). When tea seedlings were exposed to theanine, LR number and length declined by 63% and 53%, respectively (Fig. 3B and C); meanwhile LR density was also significantly lower compared with the control (Fig. 3D). On the contrary, LR number increased by 104% and LR length increased by 55% under H_2_O_2_ treatment (Fig. 3A–C). However, under theanine + H_2_O_2_, LR number and length were reduced by 37% and 33%, respectively, and LR density was reduced by 32% compared with that under CK (Fig. 3A–D). Thus, the results indicated that theanine inhibited the promoting effect of H_2_O_2_ on LR development in tea plants. A similar inhibiting effect of theanine on the promotion by H_2_O_2_ of LR development was also observed in the model plant *Arabidopsis* (Fig. 3E–H). These results supported the hypothesis that theanine inhibits LR development by modulating H_2_O_2_ accumulation.

### Theanine reduced reactive oxygen species accumulation in roots

**Figure 4:**
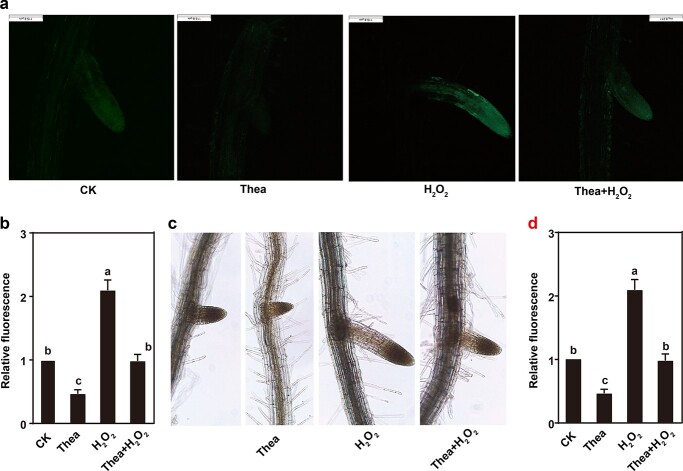
Theanine reduced H_2_O_2_ accumulation in roots. H_2_DCFDA staining (A, B) and DAB staining (C, D) for detecting H_2_O_2_ in the roots of *Arabidopsis* seedlings under control (CK), 1 mM theanine (Thea), 200 μM H_2_O_2_ (H_2_O_2_), or combination (Thea + H_2_O_2_) treatment. At least 10 samples were analyzed per treatment. Different lowercase letters above the error bars indicate significant differences by one-way ANOVA and Duncan’s multiple range test (*P* < .05). Scale bar = 0.2 mm.

To further explore the role of theanine on H_2_O_2_ accumulation, histochemical detection of ROS accumulation [2′,7′-dichlorofluorescin diacetate (H2DCFDA) staining] and H_2_O_2_ [diaminobenzidine (DAB) staining] was carried out in *Arabidopsis* roots treated with or without 1 mM theanine, 200 μM H_2_O_2_, or the combination treatment (theanine + H_2_O_2_) (Fig. 4). Compared with the control (CK), H2DCFDA staining was enhanced in LRs of seedlings treated with H_2_O_2_ but impaired in those treated with theanine (Fig. 4A and B). H_2_DCFDA fluorescence was reduced by 53% in LRs when treated with theanine and increased by 109% in those treated with H_2_O_2_ compared with the control. However, under theanine + H_2_O_2_ treatment, fluorescence was not significantly different from the control. The results of DAB staining showed a similar pattern (Fig. 4C and D). These results clearly indicated that theanine impaired H_2_O_2_ accumulation in the roots.

### Theanine regulated antioxidant enzyme activities

**Figure 5:**
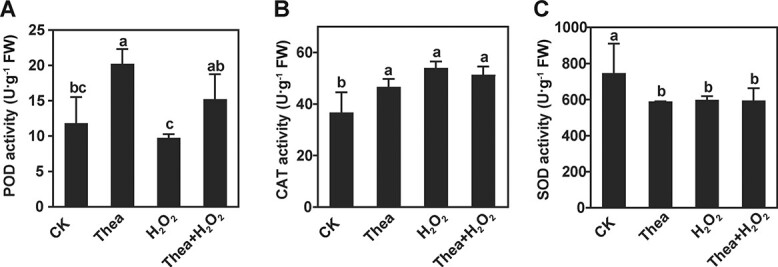
Theanine changed the activities of key enzymes in H_2_O_2_ metabolism pathway. Enzyme activity of POD (A), CAT (B), and SOD (C) in roots of tea plant under control (CK), 1 mM theanine (Thea), 200 μM H_2_O_2_ (H_2_O_2_), or combination (Thea + H_2_O_2_) treatment. Mean and standard error values were calculated from three independent experiments, and each experiment was repeated at least three times. Different lowercase letters above the error bars indicate significant differences by one-way ANOVA and Duncan’s multiple range test (*P* < .05).

To further explore the mechanism underlying the regulation of theanine on H_2_O_2_ accumulation, we measured POD, CAT, and SOD activities in tea plants. As shown in Fig. 5, POD activity was increased by 66% under theanine treatment, by 25% under theanine + H_2_O_2_ treatment, and decreased by 18% under 200 μM H_2_O_2_ treatment compared with the control (Fig. 5A). The activity of CAT was significantly increased under all treatments compared with the control (Fig. 5B), while the activity of SOD was significantly decreased under all treatments compared with the control (Fig. 5C). Given that CAT and POD catalyze the degradation of H_2_O_2_ and that SOD transfers O2^−.^ to H_2_O_2_, these results suggested that theanine represses H_2_O_2_ accumulation by affecting the activities of these antioxidant enzymes in the roots of tea plant seedlings.

### Theanine regulated the expression of genes involved in H_2_O_2_ metabolism and lateral root development in tea plants

**Figure 6:**
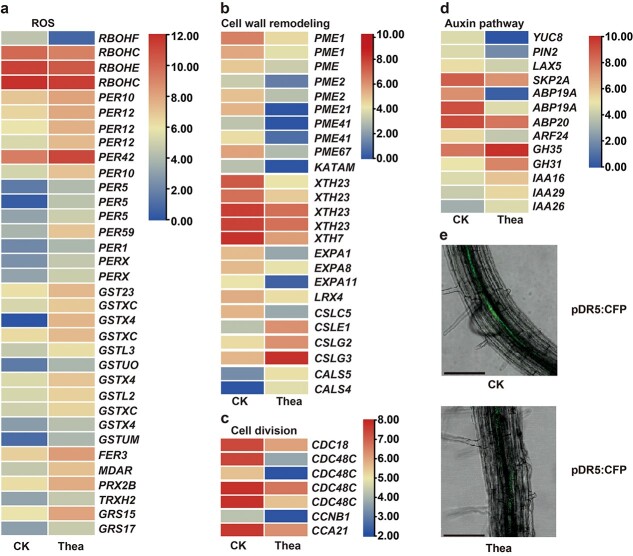
Effects of theanine on the expression of H_2_O_2_ metabolism-related and LR development-related genes in roots of tea plant seedlings. Expression of ROS metabolism-related genes (**a**), cell wall remodeling-related genes (**b**), cell division-related genes (**c**), and auxin pathway-related genes (**d**) in the roots of tea plant seedlings under control (CK) or 1 mM theanine (Thea) treatment. (**e**) Confocal images of the roots of *DR5::CFP* transgenic *Arabidopsis* seedlings. The fluorescence showed the CFP signal. Scale bar = 0.1 mm.

To reveal how theanine modulates H_2_O_2_ accumulation and LR development, we next analyzed the effects of theanine treatment on the expression of genes involved in H_2_O_2_ metabolism, cell division, cell wall remodeling, and auxin metabolism/signaling in the roots of tea plant seedlings. The expression of four *RBOH*s, including two *RBOHCs*, one *RBOHE*, and one *RBOHF*, significantly decreased under theanine treatment (Fig. 6A). In contrast, the expression of many genes encoding peroxidase, glutathione *S*-transferases (GSTs), ferredoxin, peroxiredoxin, thioredoxin, glutaredoxin, and monodehydroascorbate reductase (MDAR, an enzyme for ascorbate biosynthesis) were upregulated by theanine treatment.

Consistently with the repression of LR development by theanine treatment, the expression of many cell wall remodeling pathway genes encoding pectinesterases, xyloglucan galactosyltransferases, xyloglucan endotransglucosylase/hydrolases, expansins, and extensin was downregulated, while the expression of some genes encoding cellulose synthases was upregulated (Fig. 6B). Meanwhile, many cell division pathway genes encoding cell division control proteins and cyclins were repressed by theanine (Fig. 6C).

Theanine treatment also changed the expression of genes involved in auxin metabolism, transport and signaling (Fig. 6D). A gene encoding indole-3-pyruvate monooxygenase YUCCA8 that is important for auxin biosynthesis was downregulated. However, two genes encoding indole-3-acetic acid-amido synthetases, which inactivate auxin, were upregulated. Genes encoding the auxin transporters auxin efflux carrier component 2 (PIN2) and auxin transporter-like protein 5 (LAX5) were repressed. Five genes encoding positive regulators of auxin signaling, including four auxin receptors (SKP2A, two ABP19as, and ABP20) and a transcription factor, auxin response factor 24 (ARF24), were also downregulated, while three genes encoding auxin-responsive proteins, IAA16, IAA26, and IAA29, negative regulators of auxin signaling, were upregulated. We then treated *DR5::CFP* transgenic *Arabidopsis* seedlings with 1 mM theanine. The results showed that theanine treatment greatly decreased the fluorescence in the roots of *DR5::CFP* seedlings (Fig. 6E), further verifying that theanine reduced auxin level in the roots.

## Discussion

Tea is a perennial economic crop, and the growth state of its root system is closely related to shoot growth and the lifespan of tea plants. The root system of tea plants comprises a PR, LRs, and root hairs, and plays an integral role in anchoring the plant, absorbing water and nutrients, and synthesizing and storing metabolites [29]. Theanine, one of the most characteristic secondary metabolites in tea plants, is largely synthesized in tea roots. Theanine may act as a N reservoir in tea plants, which makes tea plants tolerant of high concentrations of ammonia [4]. However, solid evidence for this role of theanine had not yet been provided, leaving the function of theanine in the root still elusive. In this study, we demonstrated that theanine likely regulates LR development by inhibiting H_2_O_2_ accumulation.

N is one of the most critical macronutrients for plant growth and development. N is absorbed from soils by plant roots, and its availability plays significant regulatory roles in root development to control N uptake [21,30]. N not only acts as a signaling molecule, but it also modulates the levels of other signals, including amino acids [22,23], H_2_O_2_ [31], and auxin [24], to regulate LR development in plants.

In tea plants, N level and forms were shown to modulate the levels of amino acids (including theanine), ROS, flavonols, and auxin in the roots (Fig. 1B) [16–19]. Under N deficiency, the contents of ROS (including H_2_O_2_) and auxin were shown to be increased, and the content of theanine decreased, in the roots of tea plants. In these studies, LR development was also shown to be promoted by N deficiency. It is noteworthy that the promoting role of N deficiency in LR development was probably not performed by a low level of external N (NH_4_^+^ or NO_3_^−^), given that zero N (0 N) was used as the N deficiency treatment in these studies. This led us to propose that other signals, such as H_2_O_2_, auxin, and theanine, play critical roles in regulating LR development in tea plants. Many studies have revealed the positive roles of auxin and H_2_O_2_ in promoting LR development [32–34]. Here, we observed a negative role of exogenous applied theanine in LR development by modulating H_2_O_2_ in tea plants. These results implied a developmental role of theanine in tea plants, and also provided novel insights into how tea plants adjust LR development in response to N status.

In our recent study, theanine was found to have a positive role in salt stress resistance in the shoot of tea plants by modulating H_2_O_2_ accumulation [35]. In this study, exogenous theanine changed gene expression in roots, and especially altered genes encoding proteins related to extracellular region/apoplast, oxidoreductase activity, oxidation–reduction process, and H_2_O_2_ metabolic process (Fig. 2A–C). Consistently, exogenous theanine treatment reduced H_2_O_2_ content in the roots (Fig. 2D). Furthermore, H_2_O_2_ treatment significantly promoted LR development in tea plants and *Arabidopsis*, while the promoting role of H_2_O_2_ was abolished by theanine when plants were co-treated with theanine and H_2_O_2_ (Fig. 3). These results suggested that theanine also modulates H_2_O_2_ accumulation in tea plant roots, which plays a negative role in LR development.

How theanine modulates H_2_O_2_ accumulation is still a question. The DEGs in response to theanine treatment were specifically enriched in oxidoreductase activity, oxidation–reduction process, and H_2_O_2_ metabolic process, strongly suggesting that theanine regulates the expression of genes in the redox pathway in tea plant roots. However, how the specificity of theanine in regulating gene expression was achieved is totally unknown. We mixed theanine with H_2_O_2_ in a tube and did not observe any change of H_2_O_2_ content (unpublished data), suggesting that theanine is not an antioxidant. Possibly theanine functions as a signal to regulate gene expression by acting with components in the redox signaling pathway.

It is noteworthy that the concentration of exogenously applied theanine was much lower than that in root cells of tea plants. We observed that 0.1 and 1 mM exogenous theanine already significantly inhibited LR development (Fig. 1). However, the theanine content in the roots of tea plants was >10% of dry weight (Supplementary Data Fig. S3). This content was close to 170 mM in terms of concentration. We also showed that 0.1 and 1 mM exogenous theanine did not significantly changed theanine contents in the roots (Supplementary Data Fig. S3). It is probably not total theanine that regulates LR development.

LR founder cells are initiated in the pericycle of PRs [25]. Interestingly, theanine is probably also synthesized in the pericycle cells of tea plant roots, given that *CsTSI* is expressed mainly in these cells in tea plant roots [6]. Theanine accumulation in tea plant roots seems also to occur mainly in the pericycle [36]. These founder cells respond to H_2_O_2_ and auxin signals to form the LR primordium. Indeed, we observed the *DR5::CFP* signal in the pericycle of tea plant roots and the signal was much lower under theanine treatment (Fig. 6E). Thus, the cells in which theanine is biosynthesized and accumulates overlap with the cells from which LRs initiate.

**Figure 7:**
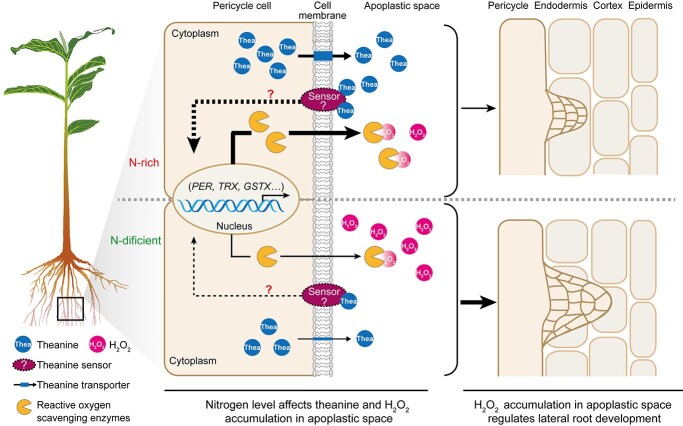
A proposed model for the involvement of theanine in regulation of LY development in response to N levels in tea plants. Under N-rich conditons, more theanine is syntheized in the pericycle and is exported to the apoplanstic space for xylem loading. The theanine in the apoplastic space is probably sensed by a plasma membrane-localized sensor, which may elicit a signal to the nucleus to induce the expression of genes encoding reactive oxygen scavenging enzymes. These enzymes are secreted into the apoplastic space of pericycle cells to scavenge H_2_O_2_, which represses the development of LRs. Under N deficiency, less theanine is synthsized and exported, and more H_2_O_2_ accumulates in the apoplastic space. The accumulated H_2_O_2_ promotes the development of LRs.

We know that root-synthesized theanine is transported to the shoots via the xylem pathway [4,5]. Before xylem loading, theanine needs to be exported to the apoplastic region [5,37]. One can imagine that the concentration of theanine in the apoplastic region of pericycle cells is dynamic in response to N status. For example, it is likely that more theanine is exported to the apoplastic region under N-rich conditions compared with that under N-deficient conditions. Thus, the dynamic concentration of apoplastic theanine could be a signal. It is likely that the theanine exported to the apoplastic region of the pericycle plays a signaling role in LR development (Fig. 7).

CsTSI is a conserved GSI-like protein [6]. In *A. nidulans*, mutation of *fluG*, the first identified GSI-like gene, disrupted the programmed induction of asexual sporulation and resulted in aerial growth and no conidial production [11]. The phenotype of the *fluG* mutant was suppressed when the mutant was grown next to wild-type colonies, suggesting that FluG synthesizes an extracellular signal directing asexual sporulation. GSI-like proteins in *Arabidopsis* and *Medicago* are NodGS, MtGSIa, and MtGSIb [12,13]. Consistent with the expression of *CsTSI*, these homologs are also specifically expressed in the roots, especially in the pericycle and LR primordium in the mature zone in *Arabidopsis* and *Medicago*. It is noteworthy that the expression of *CsTSI* and its homologs is developmentally regulated, given that they are highly expressed in the root tip, especially in the meristematic zone [6,12,13]. Interestingly, knockdown of NodGS by RNAi in *Arabidopsis* resulted in altered root development. PR development was greatly inhibited, and the LRs were longer and mainly originated from the hypocotyl/root interface zone in these RNAi lines, because of the reduction of meristematic activity and premature entry into the elongation phase [12]. These results imply that NodGS is important for maintaining meristematic activity and root patterning. However, the catalytic activity of these GSI-like proteins has not been detected, and the metabolite synthesized by these proteins remains unidentified in *A. nidulans* and *Arabidopsis*. In this study, we showed that theanine, synthesized by CsTSI, regulates LR growth in tea plants. We assume that theanine is probably a signal linking N status and root development. This also provides insights into how GSI-like proteins mediate N signaling and developmental fate.

## Materials and methods

### Plant materials, growth conditions, and experimental treatments

Seeds of 8-year-old tea plant (*Camellia sinensis* cv. ‘Longjing 43’) were harvested from the Tea Plant Germplasm Resource Garden in Guohe Town of Anhui Agricultural University. The seeds were soaked in water for 7 days to promote germination. The water was changed every day. To obtain tea seedlings for hydroponic treatment, seeds with a cracked seed coat were cultured in vermiculite at 22°C for 45 days. The hydroponic setup is shown in Supplementary Data Fig. S1, and the environmental conditions in the hydroponics experiments were controlled as previously reported [18]. Tea seedlings with a 2-cm PR were cultured in 1/2 complete nutrient solution for 14 days, and then treated with 1/2 complete nutrient solution containing 0, 0.1, 1, and 5 mM theanine, or containing 1 mM theanine with or without 200 μM H_2_O_2_ for 14 days. The complete nutrient solution formula was described previously [38].

*Arabidopsis thaliana* seeds were sterilized with 75% ethanol for 1 minute and 5% sodium hypochlorite for 10 minutes, washed with sterile water, and then cultured on ½ MS solid medium (pH 5.8). After storage in a 4°C refrigerator for 2 days, the seeds were vertically cultured in a chamber with ~120 μmol m^−2^ s^−1^ light intensity, 16/8 hours light/dark period, and 21°C/18°C day/night temperature.

Seedlings were imaged with a Canon IXUS 130 camera, and the number of LRs (>1 mm) per seedling was recorded. The lengths of LRs and PRs were measured using ImageJ. Fifteen seedlings were used to record the number of LRs and the length of LRs and PRs for each treatment. Three independent biological replicates were included for statistical analysis. Only the root mature zone was used for the following biochemical and molecular analyses.

### Reactive oxygen species detection

3,3′-Diaminobenzidine (DAB) and 2′,7′-dichlorodihydrofluorescein (H_2_DCFDA) were used to qualitatively estimate H_2_O_2_. For DAB staining, the seedlings were pretreated with ddH_2_O for 30 minutes and then dipped in 1 mg/ml fresh DAB solution in the dark at room temperature for 40. After incubation, the seedlings were rinsed with 95% (v/v) ethanol then dipped in 20% glycerol for photographing. The images were captured using a light microscope.

Similarly, for the H_2_DCFDA assay the seedlings were pretreated with ddH_2_O for 30 minutes and then dipped in 10 μM fresh H_2_DCFDA solution for 30 minutes at room temperature. After incubation, the seedlings were washed three times with ddH_2_O, and dipped in 20% glycerol for confocal analysis. The above analyses were performed according to a previously described protocol [39]. Fluorescence was detected with a Leica TCS SP8 laser confocal microscope (Wetzlar, Germany).

### Determination of activities of enzymes involved in H_2_O_2_ metabolism

The enzymes were extracted as described earlier [40]. The activities of superoxide dismutase (SOD), catalase (CAT), peroxidase (POD) and aseorbateperoxidase (APX) were measured following the instructions in kits from Nanjing Jiancheng Bioengineering Institute (Nanjing, China). Briefly, the activity of SOD was measured by the absorbance at 450 nm after 20 minutes of reaction at 37°C. One unit of SOD was defined as the amount of SOD required to produce a 50% inhibition rate. CAT-catalyzed decomposition of H_2_O_2_ can be stopped by ammonium molybdate. The remaining H_2_O_2_ forms a yellowish complex whose change of absorbance at 405 nm can be used to calculate CAT activity. One unit of CAT activity was defined as the degraded amount of H_2_O_2_ per second per milligram of protein. The POD activity was measured by monitoring the change in absorbance of catalyzed H_2_O_2_ at 420 nm. One unit of POD activity was defined as the amount of POD catalyzing 1 μg substrate per minute per milligram of protein at 37°C. Under the catalysis of APX, ascorbic acid (ASA) is oxidized to dehydroascorbate by H_2_O_2_, accompanied by decreasing absorbance at 290 nm. One unit of ASA activity was defined as 1 μmol ASA consumed per minute per milligram of protein.

### RNA isolation, Illumina sequencing, and data analysis

Total RNA was extracted using an RNAprep Pure Plant Plus Kit (Tiangen, Beijing, China). A total of 12 mRNA libraries were constructed and sequenced using the Illumina NextSeq 500 platform. Clean reads were obtained by removing adaptor sequences and filtering with HISAT2, and were then mapped onto the tea plant reference genome [7]. All genes that assembled into the genome were counted using HTSeq, and their expression levels were standardized by the FPKM (fragments per kilobase of transcript per million mapped reads) method. Genes with log_2_ |fold change| > 1 and *P*-value <.05 were identified as differentially expressed genes (DEGs) by DESeq. GO (Gene Ontology) and KEGG (Kyoto Encyclopedia of Genes and Genomes) enrichment analyses of DEGs were carried out to predict the main function and enrichment degree of genes in specific metabolic pathways.

### Gene expression analysis

The PrimeScript RT Reagent Kit was used to synthesize cDNA and TransStart Tip Green qPCR SuperMix was used for quantitative real-time PCR (qRT–PCR) on a Bio-Rad CFX96. *CsGAPDH* was used as the internal reference. Each assay was performed with three biological replicates. The primers are listed in Supplementary Data Table S1.

### Confocal microscopy

*DR5*::*CFP* transgenic seeds were provided by Professor Wenbiao Shen of Nanjing Agricultural University. Four-day-old seedlings were transferred and cultured for 2 days in ½ MS medium without or with 1 mM theanine. Cyan fluorescent protein (CFP) signal fluorescence in *DR5*::*CFP* transgenic *Arabidopsis* was imaged using a Leica confocal laser scanning microscope (Mannheim, Germany) with excitation at 434 nm and emission at 477 nm.

### Statistical analysis

Statistical analyses were performed using SPSS (V19.0) software. Means and standard errors were calculated with the results of three biological replicates. Data were analyzed by one-way analysis of variance (ANOVA) followed by Duncan’s multiple range test, with *P* < .05 indicating a significant difference.

## Acknowledgements

This work was supported by the National Key R&D Program of China (2021YFD1601101, 2018YFD1000601) and grants from the National Natural Science Foundation of China (32072624) and the Base of Introducing Talents for Tea Plant Biology and Quality Chemistry (D20026). We would like to thank the Tea Plant Cultivar and Germplasm Resource Garden in Guohe Town, Anhui Agricultural University, for providing tea plant material. We also thank Professor William J. Lucas of the University of California, Davis, for his suggestions on this study.

## Author contributions

Z.Z., X.W., G.X., and J.Z. conceived this project and designed the experiments. T.C., S.L., Z.C., T.Y., and S.Z. carried out the experiments and analyzed the data. T.C. and S.L. drafted the manuscript. Z.Z. finalized the manuscript. All authors read and approved the manuscript.

## Data availability

All relevant data in this study are provided in the article and its supplementary files.

## Conflict of interest

The authors declare that they have no conflict of interest.

## Supplementary data

Supplementary data are available at *Horticulture Research* online.

## Supplementary Material

Web_Material_uhac267
