# Supplementary material for: Theanine, a tea-plant-specific non-proteinogenic amino acid, is involved in the regulation of lateral root development in response to nitrogen status
Source: Hortic Res. 2022 Dec 2;10(2):uhac267. doi: 10.1093/hr/uhac267 (PMC9909507; doi:10.1093/hr/uhac267)
Supplement: Web_Material_uhac267 [file web_material_uhac267.docx]

**Theanine, a tea plant specific non-proteinogenic amino acid, is involved in the regulation of lateral root development in response to nitrogen status**

Tingting Chen^1, #^, Shijia Lin^1, #^, Ziping Chen^1, #^, Tianyuan Yang^1^, Shupei Zhang^1^, Jinsong Zhang^1^, Guohua Xu^2^, Xiaochun Wan^1^, Zhaoliang Zhang^1, *^

^1^State Key Laboratory of Tea Plant Biology and Utilization, Anhui Agricultural University, Hefei 230036, China

^2^State Key Laboratory of Crop Genetics and Germplasm Enhancement, Nanjing Agricultural University, Nanjing 210095, China

# These authors contributed equally to this work

*Correspondence: Zhaoliang Zhang (zhlzhang@ahau.edu.cn).

**
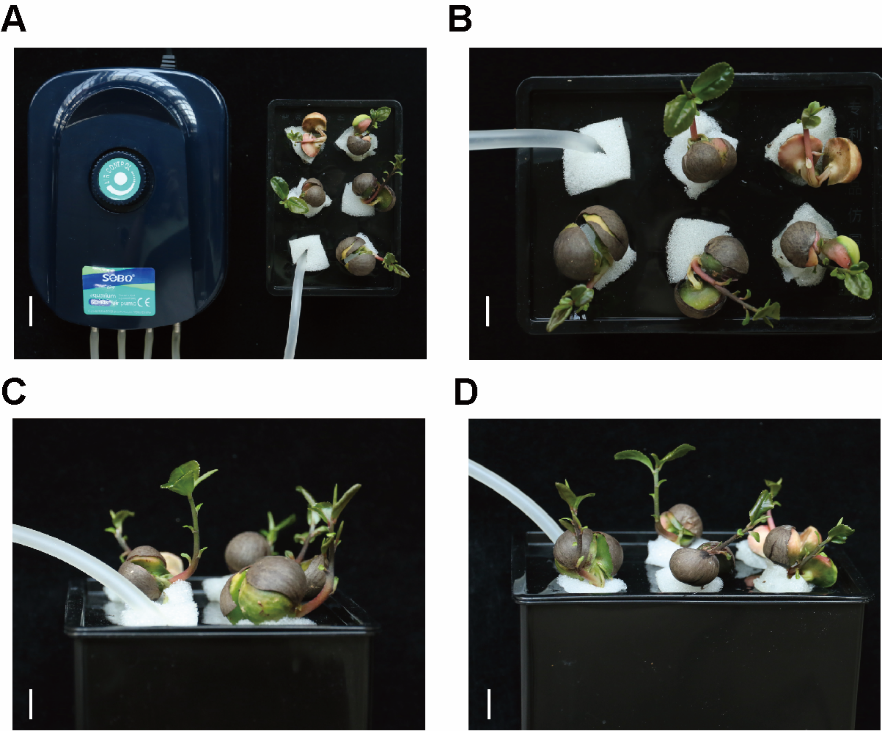
SUPPLEMENTARY FIGURES**

**Figure S1. Hydroponic culture system for tea plant seedlings. (A)** General view of hydroponic culture of tea seed seedlings. The left is an air pump, and the right is a hydroponic box and tea plant seedlings. **(B)** Top view of hydroponic system. **(C-D)** Side view of hydroponic system.

**
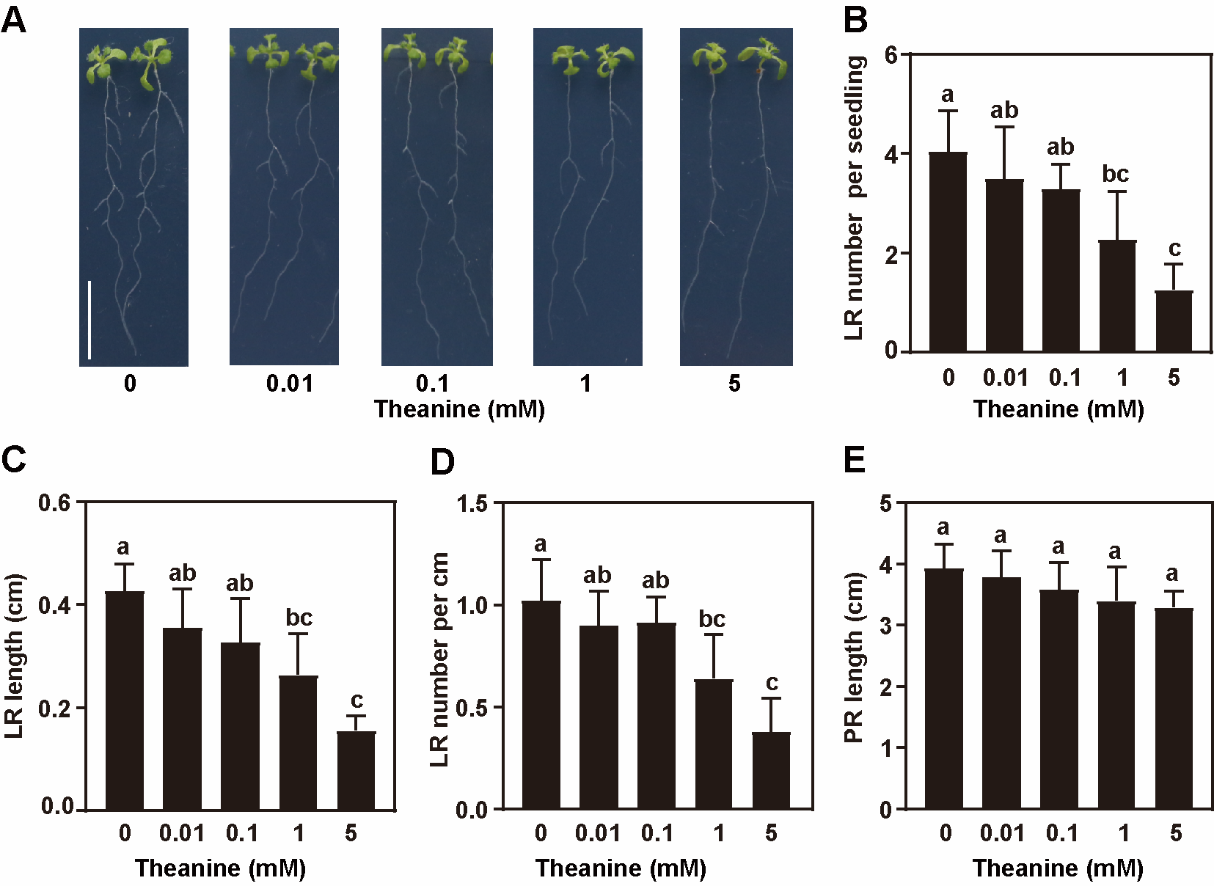
Figure S2. Effects of theanine on LR development of *Arabidopsis.* (A)** LR phenotypes of *Arabidopsis* grown in 1/2 MS with increasing theanine concentrations (0, 0.1, 1, 5 mM) for 2 weeks, scale bar = 1 cm. **(B)** The number of emerged LR (>1 mm) per seedling. **(C)** The LR length. **(D)** The emerged LR density and **(E)** PR (primary root) length. Mean and SE values are calculated from at least three independent experiments, each experiment repeated at least three times. Different lowercase letters above the error bar indicate significant differences by one-way ANOVA and Duncan’ s multiple range test (P < 0.05).

**
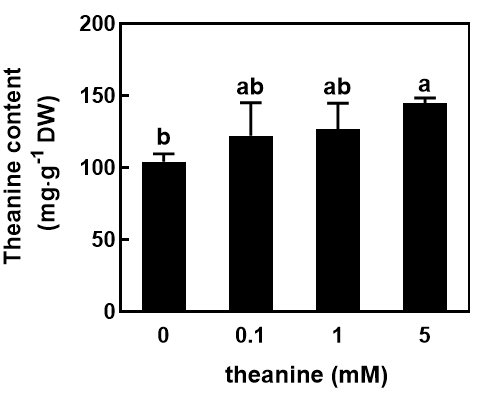
Figure S3. Theanine contents in the roots of tea plant seedlings under theanine feeding.** The seedling seedlings were treated with 0, 0.1, 1, or 5 mM theanine in the hydroponic solution.
